# Pore structure characterization and its influence on aqueous phase trapping damage in tight gas sandstone reservoirs

**DOI:** 10.1371/journal.pone.0298672

**Published:** 2024-04-26

**Authors:** Qing Guo, Xiaojia Lu, Tao Liu, Mengtao Yang, Kai Wang, Yande Zhao, Liang Tao

**Affiliations:** 1 BaiLie School of Petroleum Engineering, Lanzhou City University, Lanzhou, China; 2 The NO.6 Oil Production Plant, PetroChina Changqing Oilfield Company, Dingbian, China; 3 PetroChina Sales Shaanxi Branch, Xi’an, China; 4 Oil and Gas Technology Research Institute Changqing Oilfield Company, Petrochina Company Limited, Xi’an, China; Jeddah University: University of Jeddah, SAUDI ARABIA

## Abstract

Aqueous phase trapping (APT), which is one of the most prominent damages, seriously restricts the natural gas production in tight gas sandstone with low permeability. Pore size and microscopic pore structures are the most important factors to determine the water blocking damage. In this paper, 9 core samples from tight gas sandstone with various physical properties were employed, and the pore size distribution (PSD) of the core samples were investigated by high pressure mercury intrusion tests (HPMI). Results showed that the porosity of core samples ranges from 5.68% to 13.7%, and the permeability ranges from 0.00456 to 7.86 mD, which is a typical tight reservoir with strong heterogeneity. According to the HPMI capillary curve, the cores can be divided into two types: Type I and Type II, and the pore sizes of type I are larger than that of type II. Fractal distributions were obtained using HPMI data to further determine the pore structure characteristics of tight reservoirs. The pore structures of tight sandstones display the multifractal fractal feature: D_1_ corresponding to macro-pores, and D_2_ corresponding to fractal dimension of micro-pores. Furthermore, APT damage was determined by the permeability recovery ratios (*K*_r_) after gas flooding tests. The correlation of *K*_r_ and PSD and fractal dimensions were jointly analyzed in tight gas sandstone. Although positive correlations between pore size parameters and the permeability recovery ratios were observed with relatively weak correlations, for those core samples with very close permeability, pore size parameters (both permeability and PSD) is inadequate in clarifying this damage. The fractal dimension can well describe the complexity and heterogeneity of flow channels in pores, which can become the determining factor to distinguish the flow capacity of tight sandstone. The D_2_ for samples of type I and type II exhibited a good negative relation with *K*_r_ with a correlation coefficient of 0.9878 and 0.7723, respectively. The significance of this finding is that for tight gas sandstone, fractal dimensions, especially the small pore fractal dimension (D_2_), can be used to predict the possible APT damage very well.

## 1. Introduction

Natural gas production from tight sandstone reservoirs has gained increasing attention in recent years due to abundant gas reserves [1–3]. However, tight sandstone characterizes low permeability, strong water-wet condition and narrow pore throats, which lead to high capillary forces [4, 5]. During the drilling process of reservoirs in oil and gas fields, working fluids in the wellbore such as drilling fluids, completion fluids, fracturing fluids, acidizing fluids, etc., intrude into the reservoir due to the capillary effect, which can increase the water saturation in the formation and further cause the decrease of gas flow capacity [6–8]. In the process of oil/gas production, due to the reservoir capillary retention effect, the intruding fluid would be hard to flow out the pore space in tight sandstone, so that the relative permeability of gas cannot be restored [9]. This phenomenon is known as the “aqueous phase trapping” (APT) or "water blocking damage”, which is one of the most prominent damages in low-permeability gas reservoirs, seriously restricting the oil and natural gas recovery in low-permeability reservoirs [3, 8, 10–12]. When the wetting phase intrudes into the reservoir, the relative permeability of oil and gas decreases significantly. If the saturation of the intruding fluid reaches about 50%, the relative permeability of oil and gas will decrease by more than 90%, which is fatal to the natural gas production in tight gas sandstone [12, 13].

The microscopic pore structure of reservoirs is a main factor to cause the water blocking damage [11, 14–17]. Microscopic pore structures mainly include pore geometry, pore size distribution, and pore connectivity, which can be obtained from the pore throat distribution and capillary parameters of tight sandstones, and the relationship between pore throat size distribution and permeability was also investigated by previous researchers [18–20]. The pore systems, which include the pore throats and the pore types, are the main controlling parameter in the fluid movement. Large variation in pores size leads to a high degree of microscopic pore structure heterogeneity in tight sandstones. Numerous researchers investigated the relationship between pore parameters (such as permeability and pore size) and water blocking damage [7, 13, 21]. Jiping et al. [3] found that the permeability damage ratio increases with the declination of permeability, but the increase of porosity. They also provided that according to Kozeny-Carman, for samples with same permeability, a higher porosity means a larger capillary pressure and consequentially results in a higher final water saturation as well as PDR.

For better describing the pore structure heterogeneity in porous media, fractal theory was proposed and developed rapidly. Fractal theory has played an important role in the study of self-similar complex systems widely existed in nature since it was proposed and developed by Mandelbrot and Wheeler [22] in the mid-1970s. In 1985, Katz and Thompson [23] found that sandstone pore space also satisfies fractal characteristics based on scanning electron microscopy and related optical data. Pape [24] and others developed a fractal model of pore space to study sandstone diagenesis. Song [25] and others explored the superiority of fractal theory in describing the pore space of low-permeability sandstone reservoirs. Lei [2] et al. developed a new model based on fractal theory to characterize fluid flow in low-permeability sandstones with complex pore structures. In recent decades, many scholars have also applied fractal theory to study the fractal characteristics of porous media and their internal mass transfer phenomena. Although pore structure heterogeneity of tight sandstone can be better described by fractal theory, the relationship between water blocking damage and fractal dimensions was rarely investigated.

In this paper, we aim to investigate the relevance of aqueous phase trapping damage and pore structures including regular pore structure parameters and fractal dimensions in tight gas sandstone. First, 9 sandstone core samples with a various physical properties were employed, and the porosity and permeability were measured. Then, pore morphology and minerals were obtained. Followed that, the pore-throat size distribution (PSD) of the core samples were investigated by high pressure mercury intrusion tests (HPMI). Fractal distributions were obtained using HPMI data to further determine the pore structure characteristics of tight reservoirs. Furthermore, APT damage was determined by the drainage degree of liquid entered core samples. Finally, the correlation of aqueous phase trapping damage and PSD and fractal dimensions were jointly analyzed in tight gas sandstone. The significance of this finding is that the APT damage in tight gas sandstone can be predicted very well by fractal dimensions and pore size distributions, contributing to take timely measures to prevent and control possible APT damage and improve oil and gas production.

## 2. Experiments and methodology

### 2.1 Core samples preparation

In this work, 9 sandstone core samples with a various physical properties were used. Core samples were drilled from tight sandstone formations in Ordos basin in China. All core samples in this work were cut into cylindrical plugs with same dimensions (diameter of 2.5 cm and length of 10 cm) to be conducted on a series of core analysis. Prior to the analyzed experiments, all core plugs were cleaned using the Soxhlet extraction method with trichloromethane as a solvent to remove the residual hydrocarbon components [26, 27]. After that, the core samples were dried in a vacuum oven at 80°C until the weight of a core remain unchanged. Then the prepared samples were cut into several segments to perform the following analysis, casting thin-section tests, SEM, HPMI, and gas flooding tests.

All the samples were obtained from Sulihe sandstone in Ordos Basin in China, and they provided by the Changqing Oilfield Company in China. Analyses of the core samples are prepared by the Reservoir physics Laboratory of the Lanzhou City University, including the porosity and absolute permeability measurements, the casting thin-sections, FE-SEM analysis, high pressure mercury intrusion tests, and gas displacement tests.

### 2.2 Apparatus and methodology

#### 2.2.1 Routine core analysis

A helium porosity measurement apparatus and a soap flowmeter were employed to determine the porosity and permeability following the National Oil and Gas Industry Standard (SY/T 5336–2006). The routine physical properties of the 9 samples were obtained based on the helium expansion method and Darcy equation, respectively. The measured gas porosity and permeability are generally considered as effective porosity and absolute permeability in current publications, which can be both regarded as effective values in this study [28, 29].

#### 2.2.2 Pore morphology analysis

The casting thin-sections were observed to analyze the pore types, pore-filling minerals and pore connectivity using the Axioskop 40 polarizing microscope manufactured by Zeiss. Meanwhile, the SEM is employed to further verify the pore types, morphology, and occurrence of the pore filling minerals [18, 26]. All samples were split into thin sheets with a thickness of 0.5 cm and then were tinted through vacuum-pressure intrusion with a blue epoxy resin to emphasize the pores. Furthermore, FE-SEM analysis were performed using a SU-3500 field emission scanning electron microscope at a temperature of 25°C. The pore size, morphology and clay characteristics were observed with magnifications ranging from 100× to 15 000× under acceleration voltage of 20 kV. Additionally, mineral compositions and clay contents of core samples were quantitatively determined using a X’Pert Pro MPD X-ray diffractometer (PANalytical B.V., Netherlands) [30].

#### 2.2.3 HPMI experiment

In this paper, the pore-throat size distribution (PSD) of the core samples were investigated by high pressure mercury intrusion tests (HPMI), which was conducted on a PoreMaster 33 porosimeter (Quantachrome Instruments of Anton Paar, The United States). During the mercury injection process, mercury, as a non-wetting phase to sandstone, cannot enter pores unless external pressure was exerted on it. Thus, mercury saturation experienced a progressive growth with increasing injection pressure and vice versa. In this study, the PoreMaster-60 automatic mercury analyzer was used. The instrument allows continuous or step pressure from vacuum to 106.47 MPa. The device includes a high-pressure station and two low-pressure stations. The low-pressure stations are used for measuring the pore size above 4 μm, and the high-pressure station is used to analyze samples with a pore size of less than 4 μm. For a core sample with a wide range of pore size, the low-pressure stations and high-pressure station can be jointly employed during the mercury intrusion tests. The experiment included the following steps [31]: (1) The core sample was machined to a standard cylinder with a length of 3 cm, and the weight and diameter were recorded. (2) The apparatus was started, and followed by checking the amount of liquid nitrogen and mercury to determine if it was sufficient. (3) The core sample was placed into a tube and sealed, and then the tube was loaded into the low-pressure station. (4) Mercury was injected into the core sample in the low-pressure station. (6) After the core sample was fully saturated in the low-pressure station, the sample was then removed and transferred into the high-pressure station. (7) After mercury intrusion, the mercury extrusion was performed, and the data were merged and saved in a software in the process of the high-pressure experiment.

The corresponding pore radius were calculated based on the Washburn method [32–34]:

$$P_{c}=\frac{2\sigma \;\cos\theta }{r}$$ (1)

where *P*_*c*_ is the capillary pressure (Pa), *σ* is the surface tension (N/m), *θ* is the contact angle (degree) and *r* is the equivalent pore radius of core samples at the *P*_c_. Here, the interfacial tension is 485 mN/m, and the contact angle of mercury is 140°. In this work, the injection pressure covered a range of 20 to approximately 30000 psi. According to the results of capillary curves, the PSD and other related parameters were obtained to synthetically evaluate the pore characteristics of reservoirs.

#### 2.2.4 Determination of APT damage using the core displacement test

**Fig 1:**
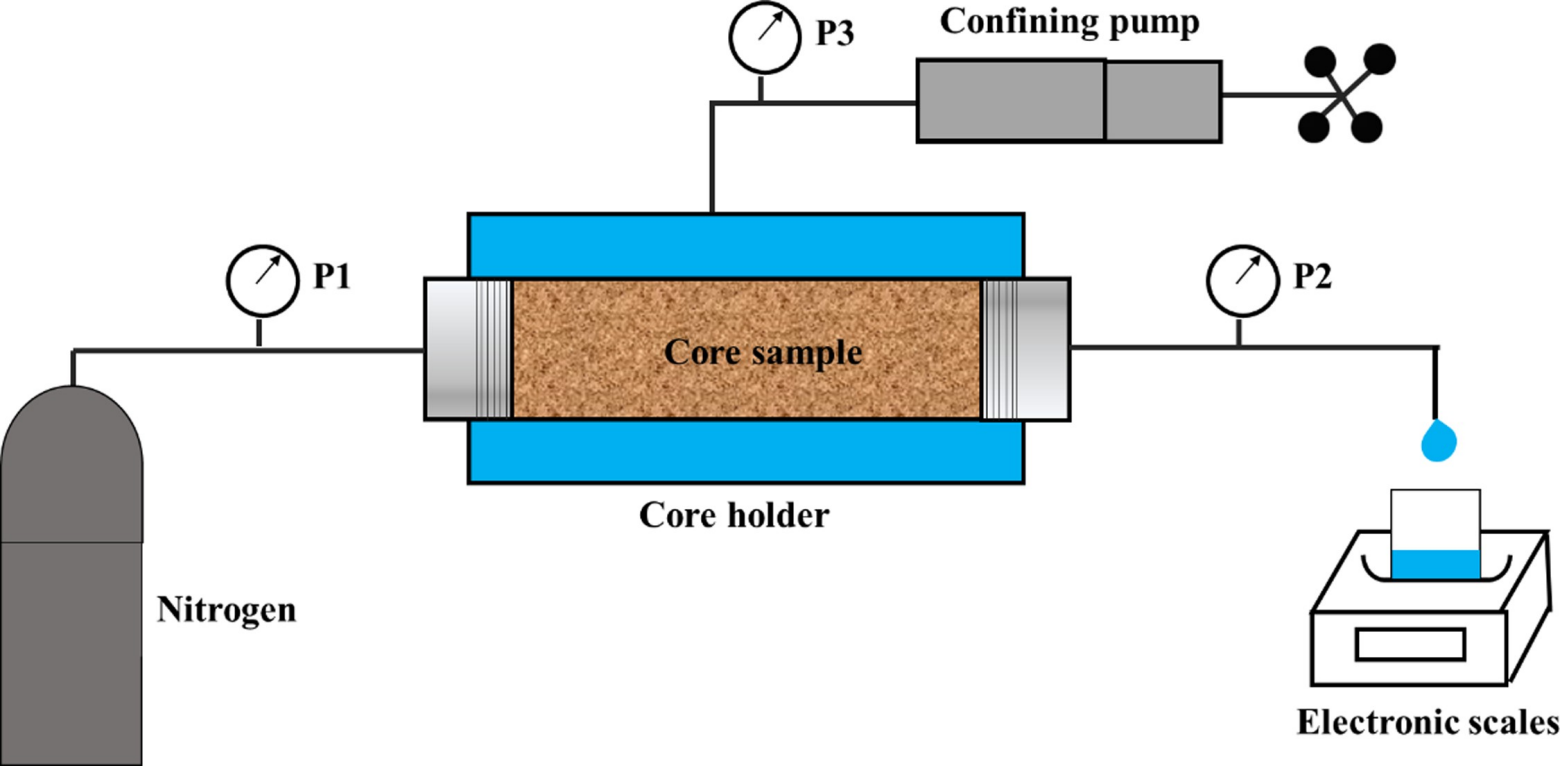
Schematic diagram of core displacement apparatus.

APT damage was determined by the drainage degree of liquid entered core samples. Permeability damage ratio is a widely used method to clarify the APT damage [8]. Consequently, we performed the core displacement tests to investigate the changes of water saturations and gas permeability of sandstone samples during the pressure drainage tests in which the samples were initially saturated. The experimental apparatus shows as Fig 1. Prior to that, core samples were firstly dried in an oven and then saturated with tested fluids under vacuum followed by collecting the physical parameters. After that, nitrogen gas was injected from inlet of the core holder by displacement pump, and then aqueous phase in core samples were removed by injected gas. Meanwhile, an electronic scale was adopted to collected the displaced liquid from the outlet end. Once the amount of liquid expelled from sandstone remained stable, the gas permeability and the weights of core plugs were immediately measured [9, 35]. Four gas flooding pressure gradients were conducted in the process of displacement step by step from 0.5 MPa to 2 MPa. Under each pressure state, core displacement test was performed until the weight of the core sample was unchanged.

In the process of gas flooding, the final gas permeability of different core samples cannot be directly used to evaluate the degree of trapping damage because of the rock heterogeneity. Therefore, the permeability recovery ratio, the ratio of gas permeability under a certain water saturation to initial gas permeability of core plug without liquids, was employed to judge the water trapping damage of different core samples [7, 10, 36]. It can be expressed by:

$$K_{r}=\frac{K_{i}}{K_{0}}$$ (2)

where, *K*_*r*_ is the permeability recovery ratio, *K*_*i*_ is the gas permeability of rock under a certain water saturation, *K*_0_ is the initial gas permeability of core plug without liquids.

In addition, irreducible water saturation (*S*_*wir*_) is also an important parameter to reveal the water block damage. Irreducible water is usually adsorbed on the mineral surface and trapped in the nano/micro pore space. For a typical tight gas sandstone, higher residual water saturation means that the more liquid phase occupy the pore space, hindering the gas flow capacity and gas recovery from formation. Herein, irreducible water saturation is defined the ratio of volume of immobile water (adsorbed/trapped water) to total pore volume.

### 2.3 Fractal analysis methodology

Fractal distributions of HPMI data were employed to further determine the pore structure characteristics of tight reservoirs. Various calculation models of fractal dimension have been established by several researchers. He and Bai et al. [37, 38] reported the calculation formula of fractal dimensions for wetting phase air. Li and Horne et al. [39] proposed a model to obtain the fractal dimensions based on the non-wetting phase mercury. It is noted that different models can describe similarly the pore structure characteristic despite various fractal dimensions obtained by these models. Herein, we employed the He and Hua models [38] to calculate the fractal dimensions of our studied core samples.

The model was derived from a power law function based on the principle of fractal geometry [22]

$$N\left(>r\right)=\int_{r}^{r_{\max}}P\left(r\right)dr=ar^{-D_{f}}$$ (3)

Where *r* is capillary diameter with characteristic linear dimension, *r*_max_ is the maximum pore radius, *N*(>*r*) is the cumulative number distribution of pores with pore radius larger than *r*, *P*(*r*) is the density function of pore size distribution, *a* is a coefficient constant. *D*_*f*_ is the fractal dimension, with the range of 2< *D*_*f*_ <3 in three dimensions.

Taking a derivation with respect to r to the both side of equation:

$$P\left(r\right)=\frac{dN\left(>r\right)}{dr}=a^{'}r^{-D_{f}-1}$$ (4)

where, *a*^’^ is a proportional constant.

The cumulative volume of pores less than *r* V(<*r*) can be expressed by the following equation:

$$V\left(<r\right)=\int_{r_{\min}}^{r}P\left(r\right)ar^{3}dr=a^{''}(r^{3-D_{f}}-r_{\min}^{3-D_{f}})$$ (5)

where *a* is a parameter related to the pore shape, rmin is the minimum pore radius of a sample.

The total pore volume of samples can be obtained through computing the integral of total pore radius of a sample:

$$V=a^{''}(r_{\max}^{3-D_{f}}-r_{\min}^{3-D_{f}})$$ (6)

Thus, the cumulative pore volume fraction less than a certain pore radius *r* can be expressed by:

$$S=\frac{V\left(<r\right)}{V}=\frac{\left(r^{3-D_{f}}-r_{\min}^{3-D_{f}}\right)}{\left(r_{\max}^{3-D_{f}}-r_{\min}^{3-D_{f}}\right)}$$ (7)

For tight sandstone, the pore size distributions exhibit a wide range. Therefore, the *r*_min_ can be deleted because of *r*_min ≪_ *r*_max_.

According to the Laplace formula [40, 41], the equation can be substituted into Eq (8):

$$S_{w}={\left(\frac{P_{c}}{P_{\min}}\right)}^{D_{f}-3}$$ (8)

where, *P*_min_ is the entry capillary pressure corresponding to the maximum pore radius, *S*_*w*_ is the wetting phase saturation at the pressure *P*_*c*_. Base on the HPMI measurements, the *S*_*w*_ can be expressed as 1-*S*_Hg_, where *S*_Hg_ is the non-wetting phase mercury saturation.

Taking logarithms on the both side of Eq (8) and substituting the *S*_Hg_, the calculation model of fractal dimension can be expressed by:

$$\log(1-S_{Hg})=(D_{f}-3)\log P_{c}-(D_{f}-3)\log P_{\min}$$ (9)

According to the Eq (8), log(1-*S*_Hg_) vs log(*P*_*c*_) were plotted and linearly fitted in a doubly logarithmic coordinates. The fractal dimension *D*_*f*_ can be obtained by the slope of the fitted line.

## 3. Results

### 3.1 Lithology and physical properties

The porosity and permeability for the 9 core samples are shown in Table 1. The porosity ranged from 5.68% to 13.7% with an average of 9.52% and permeability varied from 0.00456 to 7.86 mD (avg 1.272 mD). According to the definition by Surdam [42], the core samples in this study are characterized by lower porosity and permeability, indicating that the formation we studied is a typical tight reservoir with a high heterogeneity.

**Table 1:** The routine petrological parameters of the ten samples from target formation.

Samples	porosity [φ]/%	Permeability [k]/μm^2^	Length [L]/cm	Mass [M]/g
QS1	8.92	0.06445	5.0900	60.4690
QS3	5.81	0.04460	4.6580	54.6310
QS5	6.02	0.00456	4.9900	62.8500
LS1	8.13	0.06847	4.728	56.6427
LS2	9.71	0.08856	3.993	47.7241
LS3	5.66	0.03983	4.797	60.1262
QL21	12.32	2.82	4.812	60.3574
QL30	13.70	7.86	4.126	52.5221
QL56	12.11	0.59	4.368	51.2899

The minerals compositions of sandstone rocks are presented in Table 2. It can be seen that the quartz averagely accounts for 91%, indicating that quartz minerals are the main compositions of sandstone from Ordos basin. Carbonate minerals (calcite) in the tight sandstones mainly ranged widely from 1 to 9 wt % (average 4.8 wt %), ranking second in the minerals compositions, after quartz. The proportion of calcite mineral ranges from 1% to 9%, and clay mineral constitutes a few percent of total minerals.

**Table 2:** Minerals compositions of sandstone rocks from target formation.

Core samples	Quartz	Calcite	Kaolinite	Chlorite	Illite	Illite/smectite
QS1	88	9	0.18	1.35	0.93	0.54
QS3	89	7	0.8	1.68	1.12	0.4
QS5	97	1	0.16	1.12	0.44	0.28
LS1	91	6	0.63	1.62	0.45	0.3
LS2	94	1	0.4	2.25	1.6	0.75
LS3	93	2	1.05	1.15	2.35	0.45
QL9	91	4	0	2.6	1.95	0.45
QL21	89	4	2.05	3.32	1.32	0.31
QL30	93	3	1.25	1.65	0.75	0.35
QL56	85	6	2.25	3.51	2.52	0.72

### 3.2 Pore morphology and pore size distribution

**Fig 2:**
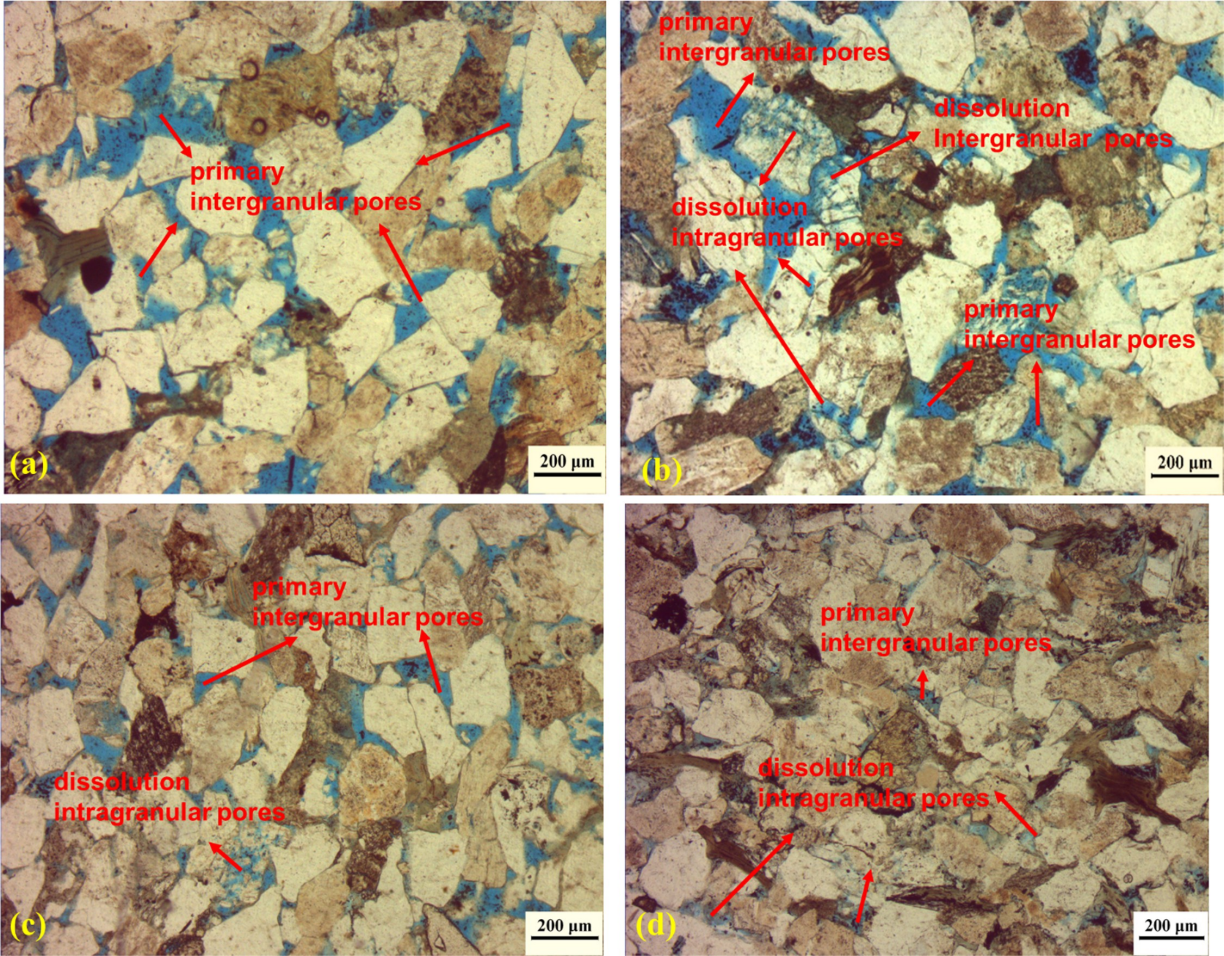
Pore types, morphology observed in the thin section analysis. Primary intergranular pores and abundant secondary dissolution pores, core with relatively high porosity. The intragranular dissolution pores due to feldspar dissolution, core with relatively low porosity. (a) Core sample of QL30; (b) Core sample of QL21; (c) Core sample of LS2; (d) Core sample of QS3.

**Fig 3:**
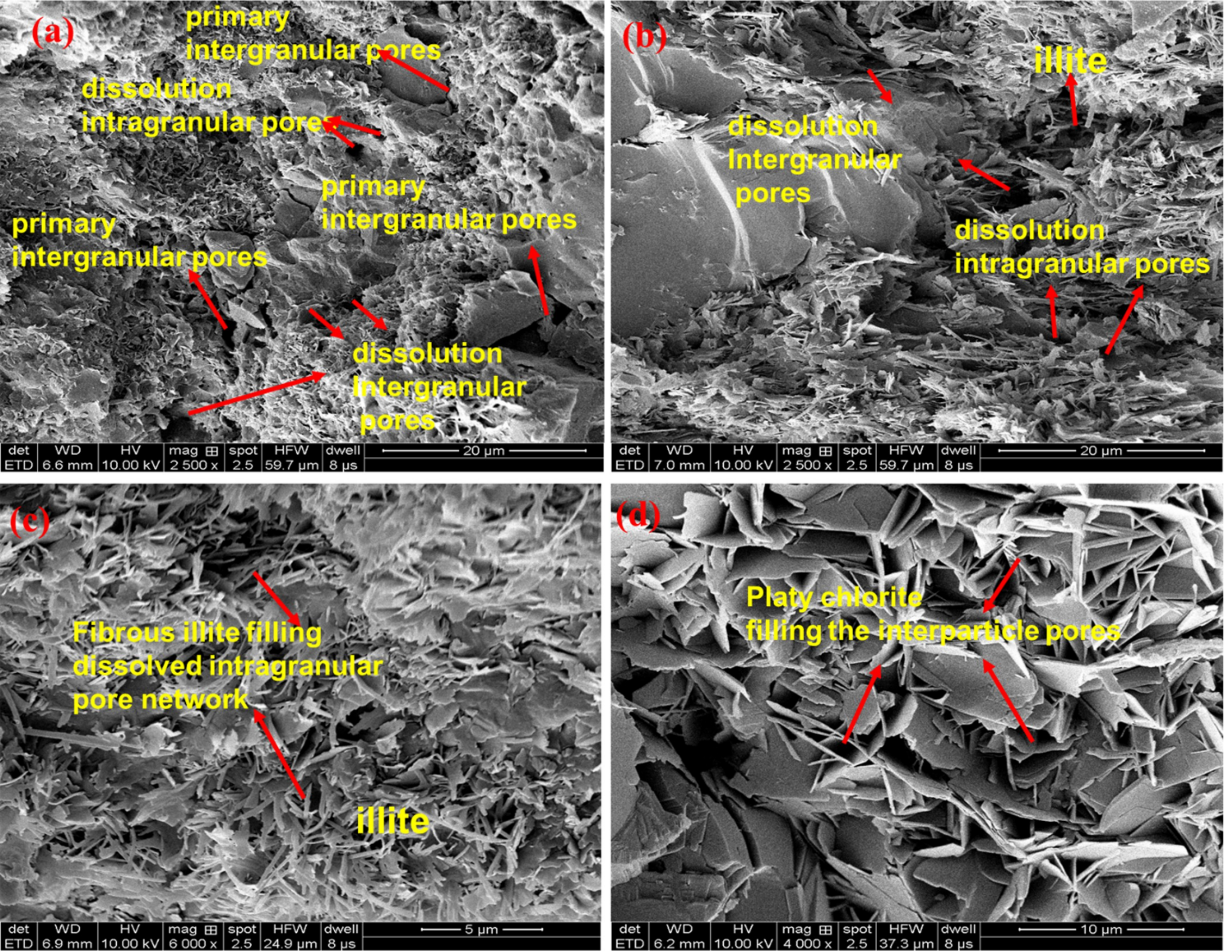
Pore types, morphology, and clay aggregations observed in SEM images. (a) Core sample of QL30; (b) Core sample of QL21; (c) Core sample of LS2; (d) Core sample of QS3.

Pore type and morphology are significant factors of the storage and fluid flowing [5, 43]. Thin section analysis and SEM analysis were applied to investigate the pore morphology characteristics of tight sandstone, showing in Figs 2 and 3, respectively. Unlike the conventional sandstone, the tight reservoirs are featured with irregular pore geometry and various pore types due to the depositional and diagenetic modifications. Four types of pores are identified in the core samples in tight sandstone: primary intergranular pores, dissolution intergranular pores, dissolution intragranular pores and intercrystalline pores [18, 20, 28]. Primary intergranular pores are commonly observed between the grains for the tight sandstones. In addition, the dissolution intergranular and intragranular pores, which experienced the dissolution of grains in acid fluids, also exist widely in tight sandstones. The intercrystalline pores are formed between the clay aggregates.

Primary intergranular pore is the main pore type, which usually exists between mineral particles with triangular or polygonal outlines, and greater pore size [28]. Fig 1(A) gives the information about the thin section analysis for the core sample QL30, showing that primary intergranular pore dominates the pore type in the sample. The dissolution intergranular pores usually appear along the edges of mineral grains and extend the pore space of intergranular pores with irregular pore shapes due to the dissolution of the feldspar, quartz, and lithic fragments. Dissolution intergranular pores can be mainly observed in the Fig 2(A) and 2(B) for core sample QL30 and QL21, indicating a positive correlation between dissolution intergranular pores and greater porosity. In addition, The intragranular dissolution pores typically result from the intraparticle dissolution in feldspar grains, which can slice and divide a mineral grain into a pore network consisting of a large amount of small-scale dissolution pores. Fig 2(A) and 2(B) from the thin section analysis and Fig 3(A) and 3(B) from the SEM analysis give the intragranular dissolution pores with various morphology. The intragranular dissolution pores is usually companied by the existence of Illite aggregates due to the dissolution of feldspar, and thus illites aggregations can be observed around intragranular dissolution pores in the SEM images (Fig 3C). Illite are generally fibrous aggregations in tight sandstones, cutting the primary pore spaces and increasing the heterogeneity. The detrital feldspars grains gradually transformed and evolved into chlorite, with growing into pore spaces (Fig 3D). Platy chlorite minerals mostly aggregates in intercrystalline pore with small sizes and poor connectivity.

Significant variations in the pore types can be found for the core samples with different porosity and permeability. For the core samples with greater porosity and permeability (QL30, QL21), pore types are dominated by the primary intergranular pores, followed by a small amount of dissolution intragranular pores (Fig 2A and 2B). For the samples with lower permeability, pore spaces is mainly contributed by the dissolution intragranular pores, because the majority of intergranular spaces are sealed by cements (Fig 2D). This finding is also consistent with previous literatures [28].

### 3.3 PSD from HPMI measurements

**Fig 4:**
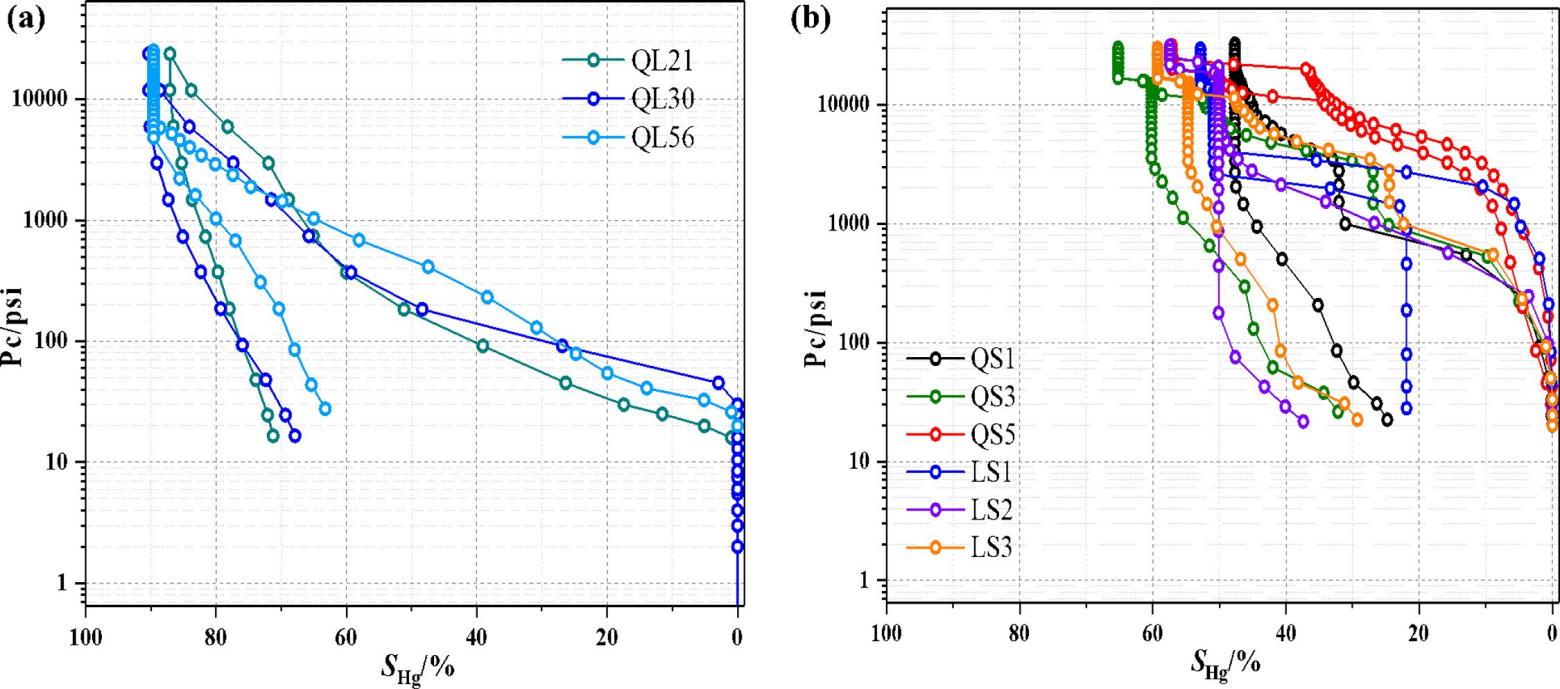
The capillary curves from HPMI of the two types. (a) Type 1: the core samples characterize a higher maximum mercury saturation (*S*_max_) with over 80%; (b) Type II: the maximum mercury saturations of the core samples approximately range from 40% to 60%.

The HPMI capillary curves of 9 core samples are shown in Fig 4. The HPMI curves present a similar pattern that mercury was gradually intruded into pores with the increasing injection pressure, while part of the mercury flowed back in the process of extrusion. According to the characteristics of the capillary curves, the 9 core samples could be categorized into two types, namely type I and type II. Core samples of type I, including samples of QL9-1, QL21, QL30, QL56, characterized a higher maximum mercury saturation (*S*_max_) with over 80%, indicating that mercury could be invaded almost all pores under high injection pressure. In comparison, the *S*_max_ of samples of type II were approximately ranged from 40% to 60%, which means that a quite partial of pores have no adequate percolation potential to allow extra mercury to intrude into.

According to the HPMI curves, the characteristic parameters were calculated and shown in Table 3. It can be seen that the threshold pressure *P*_*d*_, the point on the injection curve where mercury first entered the sample, shows a discrepancy between the two types. Type I exhibits a relatively lower *P*_*d*_, ranging from 0.110 MPa to 0.1911 MPa with an average of 0.148 MPa. For type II, the threshold pressure varies between 0.1684 to 0.3431 MPa, and the mean value is 0.2662 MPa, which is higher than that of type I. In terms of *P*_50_, which refers to the point of injection pressure where half of mercury was invaded into pores, the mean *P*_50_ of type I is just 1.923 MPa. However, for type II samples, the *P*_50_ shows quite higher, ranging from 4.137 MPa to 151.686 MPa with an average of 66.061 MPa, indicating that it is more difficult for mercury to intrude into type II tight formations than type I.

**Table 3:** Pore structure parameters of the 9 samples.

Type	Core samples	*P*_*d*_ /MPa	*P*_50_ /MPa	*r*_max_ /μm	*r*_50_ /μm	*$\bar{r}$* /μm	*S*_max_ /%	*We* /%
Type I	QL21	0.110	1.207	6.688	0.609	1.421	87.079	18.189
	QL30	0.112	1.459	2.356	0.504	0.577	90.347	24.852
	QL56	0.179	2.841	8.212	0.9213	1.7696	85.16	41.61
Type II	QS1	0.1684	-	2.6430	-	0.2689	47.68	48.01
	QS3	0.3431	59.8463	2.2538	0.0123	0.1621	65.22	46.49
	QS5	0.2966	151.6856	1.8887	0.0048	0.1029	57.13	99.60
	LS1	0.2151	28.4501	0.0383	0.0258	0.1360	52.83	56.96
	LS2	0.2308	4.1369	5.1292	0.1775	0.3564	50.13	25.41
	LS3	0.3431	86.1850	2.1854	0.0085	0.1654	55.92	47.63

**Fig 5:**
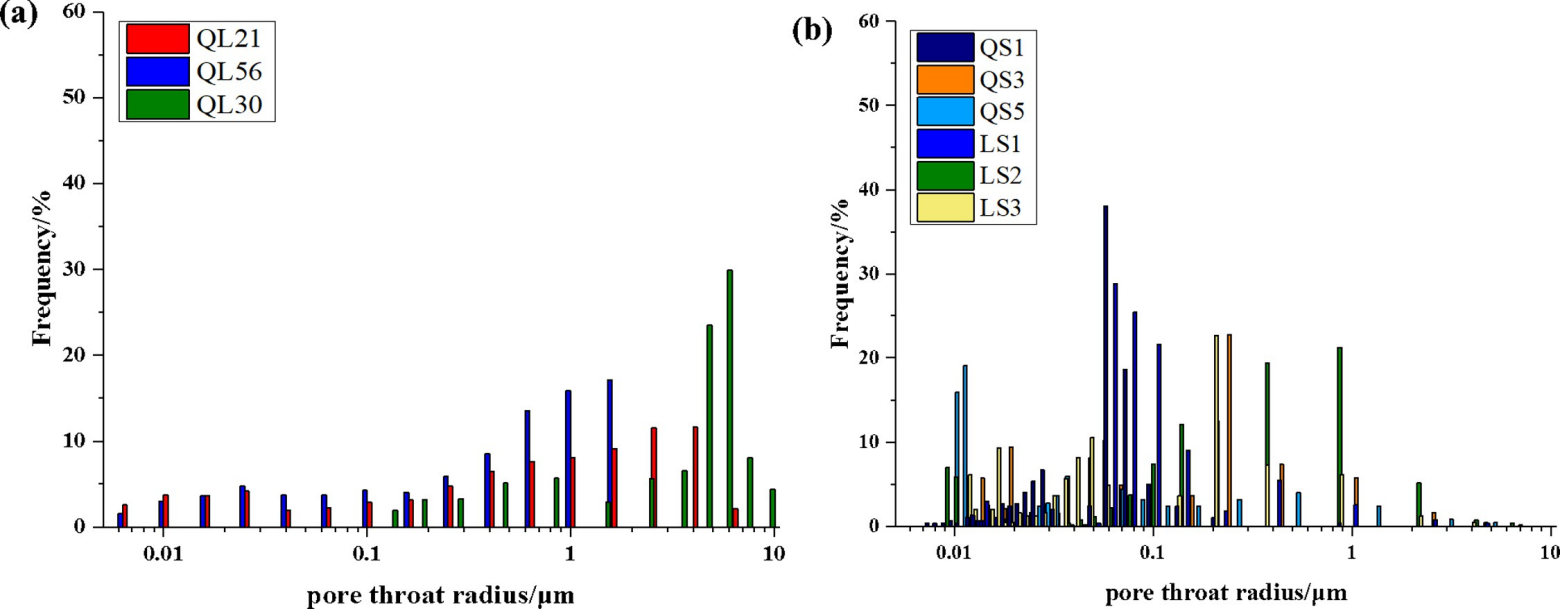
The pore size distribution from HPMI of the two types. (a) Type I: the core samples of type I exhibited a wide PSD ranging from 0.01 μm to 10 μm; (b) Type II: the pore sizes of the Type II core samples range from 0.01μm to 1μm in radius.

The conclusion could be further supported by the PSDs of 9 core samples which were obtained by results of HPMI based on the Washburn equation [27, 34]. As shown in Fig 5, obviously, the sorting of type I exhibited a wide PSD ranging from 0.01 μm to 10 μm, while the main pore radius covered radii of 0.5 to 10 μm. From the Table 3, the maximum pore size *r*_max_ of type I is in range of 2.356 μm to 8.212 μm, and the average pore sizes range from 0.577 μm to 1.7696 μm. It indicates that the core samples of type I are dominated by relatively large pores. In contrast, for the type II samples, the pore sizes ranged from 0.01μm to 1μm in radius, and the main PSD spanned a radii of 0.05∼0.5 μm with a peak of around 0.07 μm. The *r*_max_ of type II is in range of 0.0383 μm to 2.6430 μm, and the average pore sizes range from 0.1029 μm to 0.3564 μm. From the perspective of PSDs, the type I shows larger pore-throat size than type II. The HPMI results indicate that the pore structure complexity increases and the pore sorting gets worse with decreasing permeability. In addition, for the core samples with greater permeability, the pore size distributions show a wide range. Reversely, the pore sizes are smaller for the core samples with lower permeability [34]. The core samples in this study can be divided into two types according to the HPMI results and pore morphology. The tight sandstone reservoirs with similar permeability generally share similar pore structure characteristics [28].

### 3.4 Fractal dimensions from HPMI

**Fig 6:**
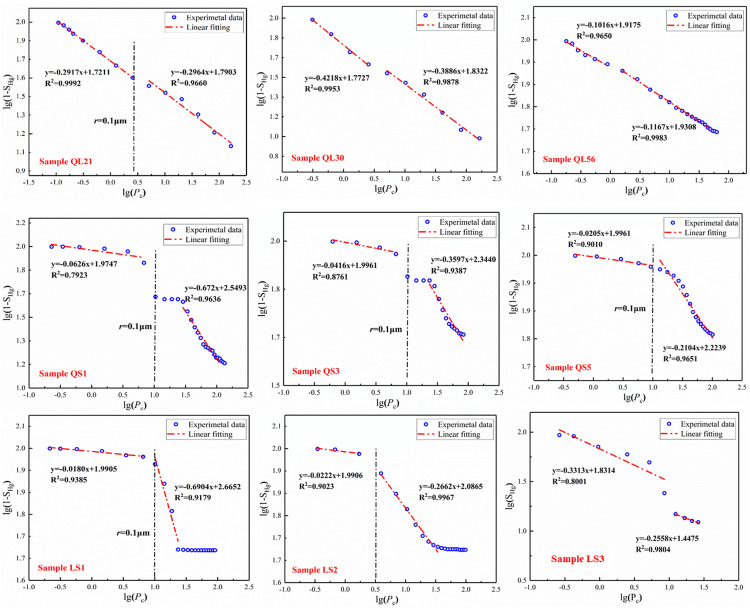
The multi-fractal characteristics of the core samples for type I and type II.

According to the fractal model, the logarithmic capillary pressure log(*P*_*c*_) and logarithmic wetting phase saturation log(1-*S*_Hg_) were plotted in Fig 6 for the 9 samples based on the data of mercury intrusion. The experimental scattered points were fitted and the fractal dimensions were calculated through the slopes of the linear fitting. It is evident that the scatter distribution can be divided into two segments, and the correlation coefficients of the two regions are high with over 0.9 of the Square-R of fitting curves for nearly all samples, indicating that these tight gas sandstone samples are generally fractal and can be characterized by using the theories of fractal geometry [25, 44, 45]. Besides, Fig 6 shows that the experimental data can be divided two segments with different slopes by a certain inflection point. The left segment of inflection point was the low-pressure region which disclosed the larger pores, while the right segment described the high-pressure region dominated by the micropores. The results of fractal dimensions for the two different segments were listed in Table 4. In fact, a same conclusion was reported by Lai et al. [44] through mercury injection test and fractal analysis for 60 tight sandstone core samples, showing that the curve of log (S_Hg_) versus log(P_c_) breaks into two segments at a particular position with various slopes for the two sections. On the basis of conclusion from Song et al. [25], the pore structures of tight sandstones can be revealed by multi-fractal characteristics due to the double-logarithm coordination relationship between the capillary pressure (Pc, MPa) and the mercury saturation.

**Table 4:** The multi-fractal dimensions of core samples for type I and type II.

Type	Core samples	D1	D2
Type I	QL21	2.7083	2.7036
	QL30	2.5782	2.6114
	QL56	2.8984	2.8833
Type II	QS1	2.9374	2.3280
	QS3	2.9584	2.6403
	QS5	2.9795	2.7738
	LS1	2.982	2.3096
	LS2	2.9778	2.7338
	LS3	2.9703	2.6936

Interestingly, the values of inflection point for samples of type I were less than that of type II, indicating that, in the macropores region, the pore radius for samples of type I were larger than that of type II, which is in agreement with the finding of section 4.1. The left segment discloses the fractal characteristics of larger pores (D_1_), whereas the right segment provides the fractal dimensions of micropores (D_2_). We can see that the values of D_1_ for samples of type I are close to that of D_2_ ranging from 2.5782 to 2.8984 and from 2.6114 to 2.8833, respectively. For the samples of type II, the values of D_1_ are over 2.9, and always larger than D_2_ with a scope of 2.3096 to 2.7738. On the basis of previous reports [25, 46, 47], the larger pores characterized a more complicate heterogeneity than micropores. Zhang et al. [21] clarified that there are good correlations between the fractal dimensions and the physical parameters, suggesting that the fractal dimensions can be used as the index to reflect the pore structure and heterogeneity of the reservoirs.

**Fig 7:**
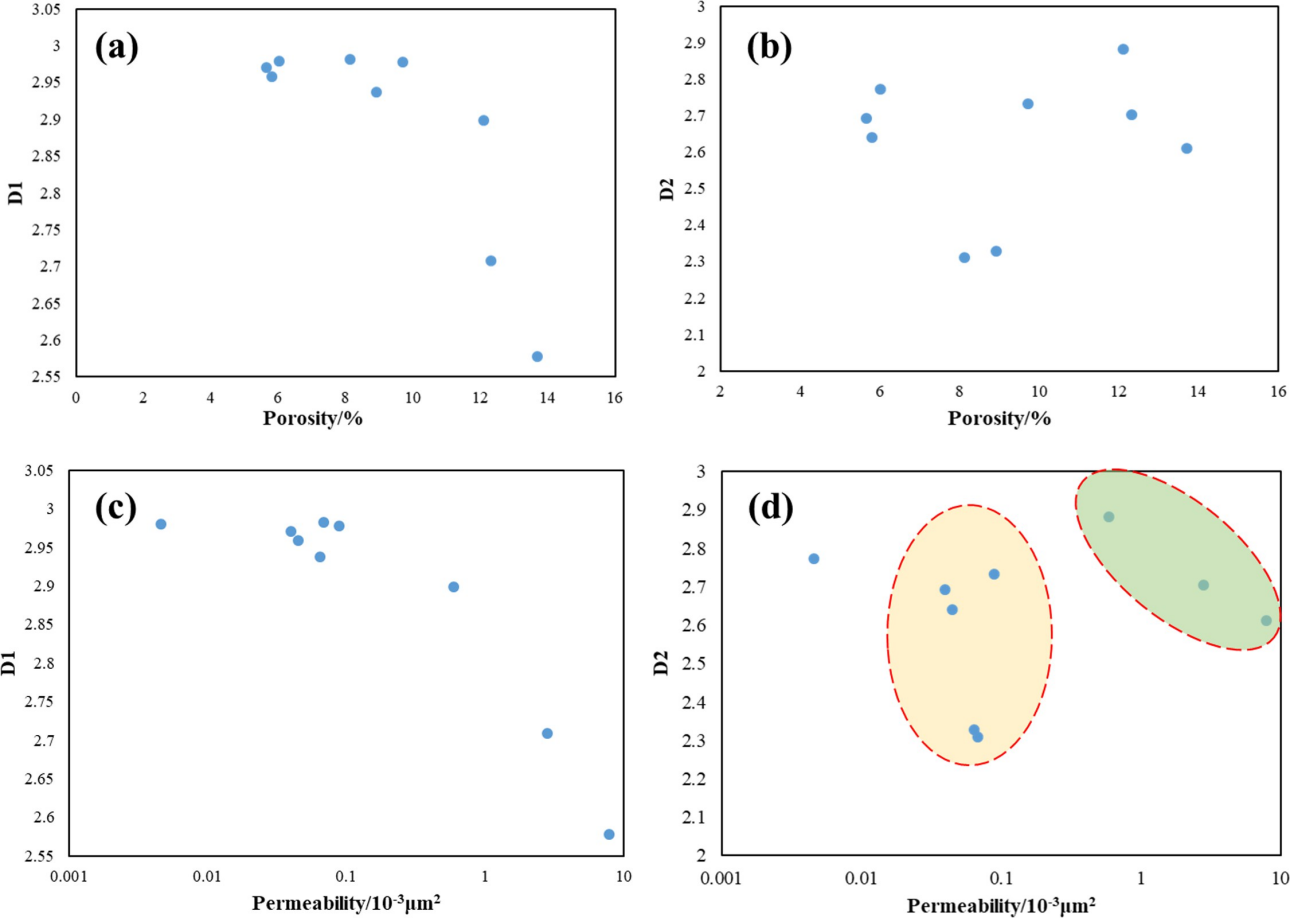
Plots showing (a) the relationship between porosity and D1; (b) the relationship between porosity and D2; (c) the relationship between permeability and D1; (d) the relationship between permeability and D2.

**Fig 7** shows the correlation between the two fractal dimensions and the porosity and permeability. Form the Fig 7A, a positive correlation between D1 and porosity of the sandstone can be seen, but there is no obvious correlation between porosity and D2. Lai et al. [44] clarified that as the cementation and compaction aggravates, the porosity of the rock decreases, which means that the more complex the pore structure, leading to the increasing D1. However, this trend shows a weak correlation when the porosity becomes smaller (about below 10% in this study), which also indicates that the porosity is not strongly affected by the homogeneity complexity of the pore structure. Fig 7(C) shows that the correlation between D1 and permeability is positive on a large scale, indicating that the smaller the core permeability, the more complex the pore structure. However, for the fractal dimension of small pores (D2), the relationship between permeability and D2 is inconsistent for core samples with different permeability, that is, when permeability is greater than 1mD, permeability increases as D2 decreases (the green region of the Fig 7(D)), while for cores with permeability less than 1mD, there is a weak correlation between permeability and D2. For the yellow region of the Fig 7(D), the permeability of core samples are very close, while their fractal dimensions are quite different, which means that even if the permeability of two cores are the same, the pore structure can be discrepant. On the basis of previous literatures [3, 10, 13, 48], the flow capacity of fluid/gas in pores is not only related to permeability, but also controlled by pore structure (fractal dimension). Therefore, both permeability and fractal dimension may contribute to the aqueous trap damage.

According to the PSD and permeability contribution, the macropores are related to the percolation capability of samples, and pores with small sizes show the storage capability of samples. Therefore, the fractal dimensions obtained from the low-pressure regions reveal the seepage properties of tight sandstone, whereas the fractal dimensions calculated from the high-pressure stage represent the storage capacity of samples [49, 50].

### 3.6 Evaluation of water blocking damage

In this section, we conducted the gas displacement tests to evaluate the water blocking damage of tight cores.

**Fig 8:**
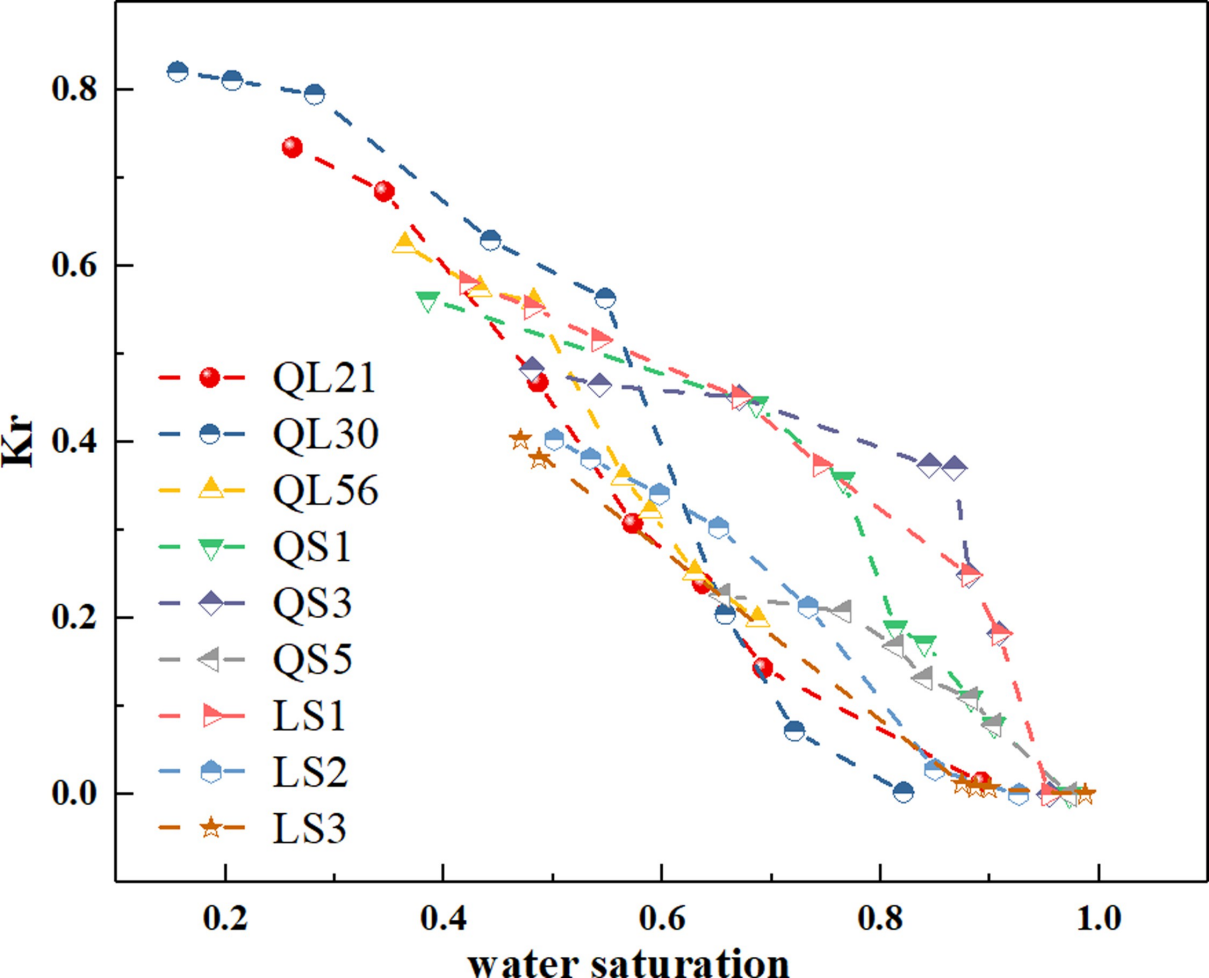
The evolution of water saturation and permeability recovery ratios during gas displacements.

Firstly, the core samples were saturated with water and theoretically the gas flow channels can be completely blocked, leading to the undetectable gas permeability. Fig 8 shows the evolution of water saturation and permeability recovery ratios (*K*_*r*_) during the drainage process. With the gas displacements, water saturations of all samples gradually decreased and the corresponding *K*_*r*_ showed an increase in the whole drainage process. Moreover, the permeability increased with a higher rate in the initial stage of gas flooding, while, as the drainage went on, the permeability remained stable. According to the results of gas flooding tests, the irreducible water saturations and the corresponding *K*_r_ were collected in Table 5. It is clear that the irreducible water saturations of the samples of type I are almost below 0.4 after gas flooding, which are lower than that of samples of type II with the range of 0.3852 to 0.6548. In terms of the permeability recovery ratio, for the core samples of type I, the values are over 0.6 at the end of gas displacement, which means that a significant number of pores were released and then occupied by gas again, suggesting that the water blocking damages are relatively low, whereas the *K*_r_ that corresponding to the irreducible water saturation for samples of type II are less than 0.6 with the minimum of 0.2262.

**Table 5:** The irreducible water saturation and gas permeability recovery ratio of water-saturated core samples after gas flooding.

Type	Core samples	Irreducible water saturation	Gas permeability recovery ratio (*K*_r_)
Type I	QL21	0.2612	0.7352
	QL30	0.1560	0.8214
	QL56	0.3640	0.6240
Type II	QS1	0.3852	0.5632
	QS3	0.4802	0.4834
	QS5	0.6548	0.2262
	LS1	0.4207	0.5813
	LS2	0.5010	0.4030
	LS3	0.4870	0.3814

For tight sandstone, liquid phase exists in micropores and larger pores as bounded fluid and movable fluid, respectively. The movable fluids can be drained by gas, and the corresponding gas permeability can recover again. Therefore, the damage of movable fluids to gas permeability is temporary. However, for the irreducible fluids, it was an challenge to remove them from pores because irreducible fluids were bounded in the smaller pores and the surface of macropores through capillary force and various surface forces, respectively. Therefore, the bounded fluids occupied the pore-throats that originally contribute to the gas flow. It means that the reservoir can suffer irreversible gas permeability damage due to bounded fluids. Herein, the irreducible water saturation and the corresponding *K*_r_ were used to evaluate the degree of water blocking damage of core samples.

## 4. Discussion

### 4.1 Effect of PSD on physical properties of tight gas sandstone

According to the HPMI measurement, the incremental and cumulative mercury saturation reflect the storage capacity of a tight sandstone sample. As shown in Fig 5, the PSDs cover a wide range from nanopores to micropores, indicating that various sizes of pores controlled the storage capability of tight reservoirs. In contrast, the percolation capability shows a distinct discrepancy. Here, we used incremental permeability contribution Δ*K*_*j*_ and cumulative permeability contribution *K*_*j*_ to disclose the percolation potential of samples based on the transformation of the Kozeny−Carman equation, shown in the following equations:

$$\Delta K_{j}=\frac{\int_{S_{j}}^{S_{j+1}}r_{i}^{2}dS}{\int_{0}^{S_{\max}}r_{i}^{2}dS}$$ (10a)

$$K_{j}=\int_{0}^{S_{\max}}\Delta K_{j}$$ (10b)

where *r*_*i*_ is the pore throat radius (μm); *S*_*j*_ is the mercury saturation corresponding to the pore throat radius (%).

**Fig 9:**
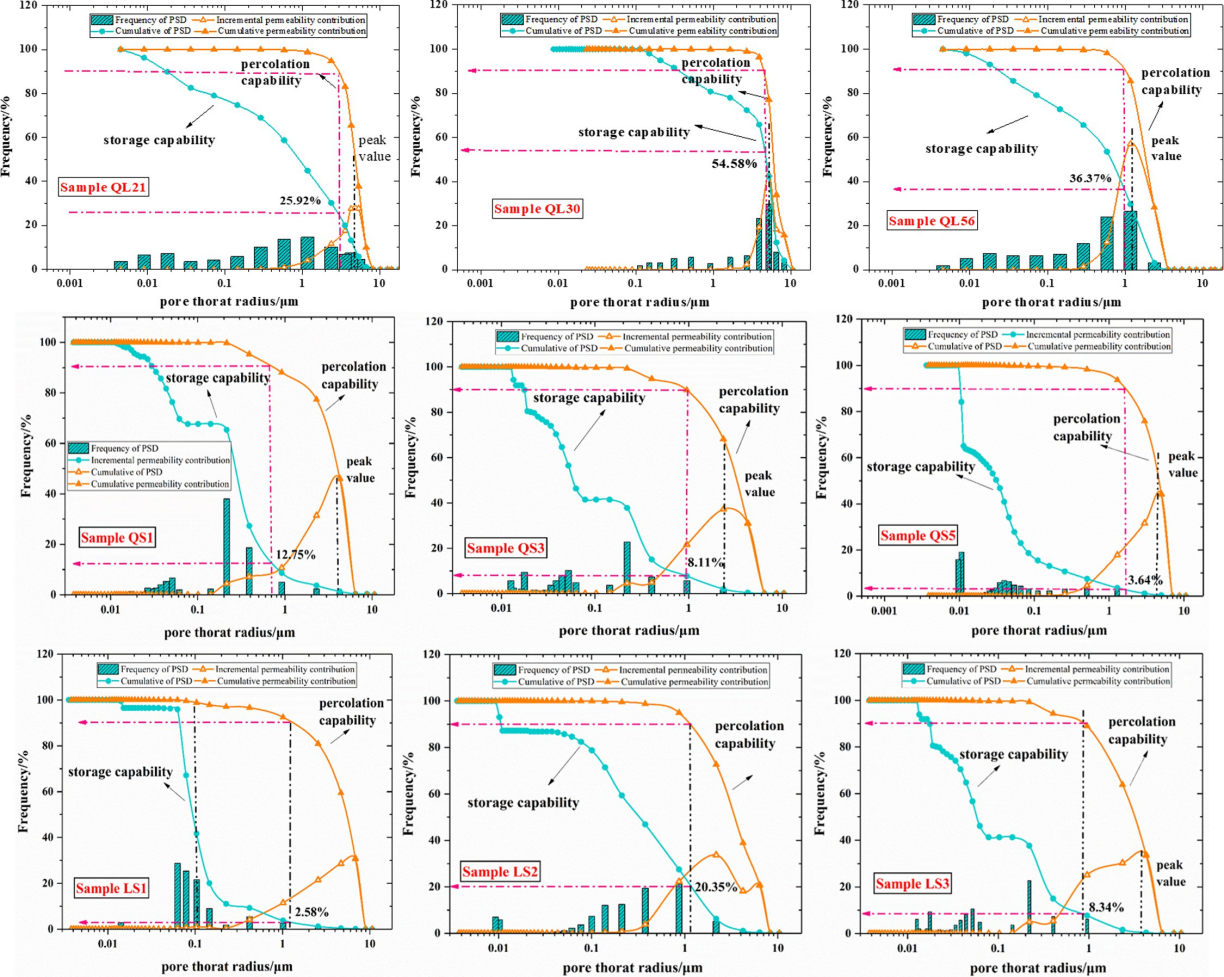
Incremental permeability contribution Δ*K*_*j*_ and cumulative permeability contribution *K*_*j*_.

The results of Δ*K*_*j*_ and *K*_*j*_ of each sample were plotted in a same graph with the incremental and cumulative PSDs. As shown in Fig 9, the incremental permeability distribution curves of samples exhibit an obvious peak at relatively larger pore radius, suggesting that the percolation potential of samples is dominated by larger pores. Furthermore, we set a line which was perpendicular to the *X*-axis at the peak value and intersect the curves of cumulative PSD and cumulative permeability contribution. The intersections of the vertical line with the two curves stand for the storage capability and percolation potential of one sample at the peak value. In the same way, we also obtained the cumulative mercury saturation and the corresponding pore radius at cumulative permeability contribution of 90%. As shown in Table 6, the *K*_*j*_ of type I samples at the peak value varied from 62.15% to 85.45% with an average of 72.36%, while for the type II samples, the *K*_*j*_ shows relatively lower, ranging from 30.79% to 72.65% (avg. 57.61%). In terms of contributions of pore throats to storage capability of samples, the cumulative mercury saturations at peak values of type I samples with a range of 6.95% to 38.15% are also larger than that of type II samples (from 0.15% to 6.22%). It means that core samples of type I have better storage capability and seepage capacity than samples of type II.

**Table 6:** The cumulative mercury saturation and the corresponding pore radius at cumulative permeability contribution of 90%.

Type	Core samples	Peak value			*K*_*j*_ *=* 90%	
		Pore radius (μm)	*K*_*j*_ (%)	*S*_Hg_ (%)	*r*_*k*-90_ (μm)	*S*_Hg_ (%)
Type I	QL21	4.76	65.43	13.24	3.099	25.92
	QL30	5.449	76.41	6.95	5.412	54.58
	QL56	2.358	85.45	26.74	1.003	36.37
Type II	QS1	4.250	45.98	1.33	0.691	12.75
	QS3	2.349	68.21	2.03	0.952	8.11
	QS5	1.252	64.19	1.87	0.325	3.64
	LS1	6.837	30.79	0.15	1.267	2.58
	LS2	2.172	72.65	6.22	1.170	20.35
	LS3	2.349	63.86	1.62	0.86	8.34

When the cumulative permeability contribution reached to 90%, for the samples of type I, the corresponding pore radius, namely *r*_*k*-90_, were in the range of 1.003–5.642 μm. It means that the larger pores over 1 μm contribute to 90% of percolation capability of samples. However, the figures for type II samples covered radius of 0.325 μm to 1.267 μm, which is lower than that of type I, suggesting that the permeability potential is dominated by a relatively lower pores. Additionally, when the cumulative permeability contributions are 90%, the corresponding cumulative mercury saturations (*S*_k-90_) of type I samples range from 25.92% to 54.58%, which also larger than the type II samples with a range of 2.58%-20.35%. Hence, it is appropriate to say that, compared to the samples of type I, more pores that contribute to storage capability for samples of type II fail to dominate the percolation capability.

### 4.2 Relation between PSD and phase trapping damage

**Fig 10:**
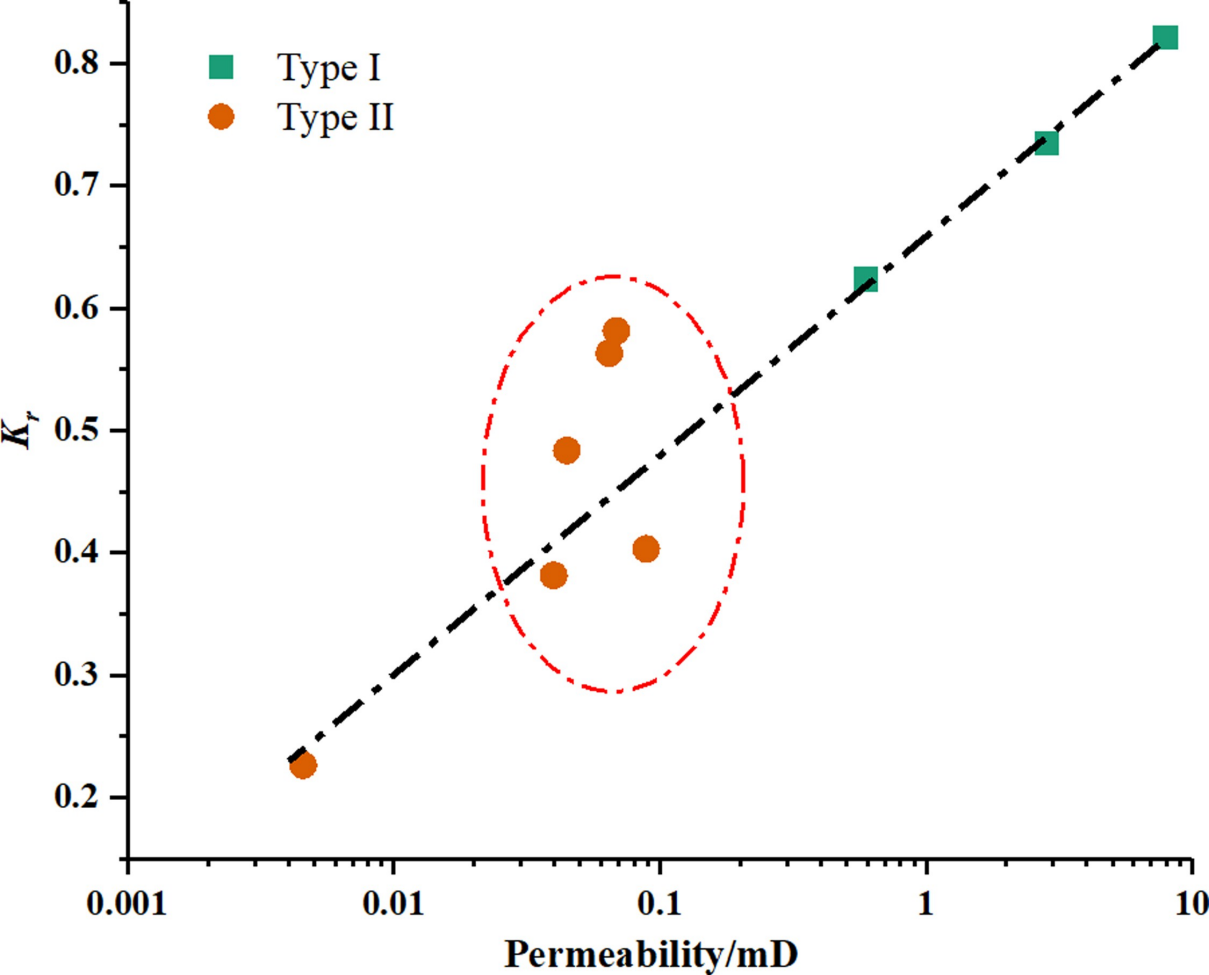
The relationship between the permeability and permeability recovery ratios *K*_r_.

Fig 10 gives the relationship between the permeability and APT damage. Results shows that there is a strong correlation within a wide range of permeability, suggesting that the value of permeability can determine and predict the APT damage of tight gas sandstones to a certain extent. For instance, for two core samples with different in order of magnitude, it can be easily predicted the possible APT damage can be very different. However, for the core samples with minor difference in permeability, the APT damage would be ambiguous and unpredictable, seeing the values in the orange region in Fig 10. It suggests permeability is not sufficient to describe the discrepancy in fluid flow in porous media, especially for the tight gas sandstones.

**Fig 11:**
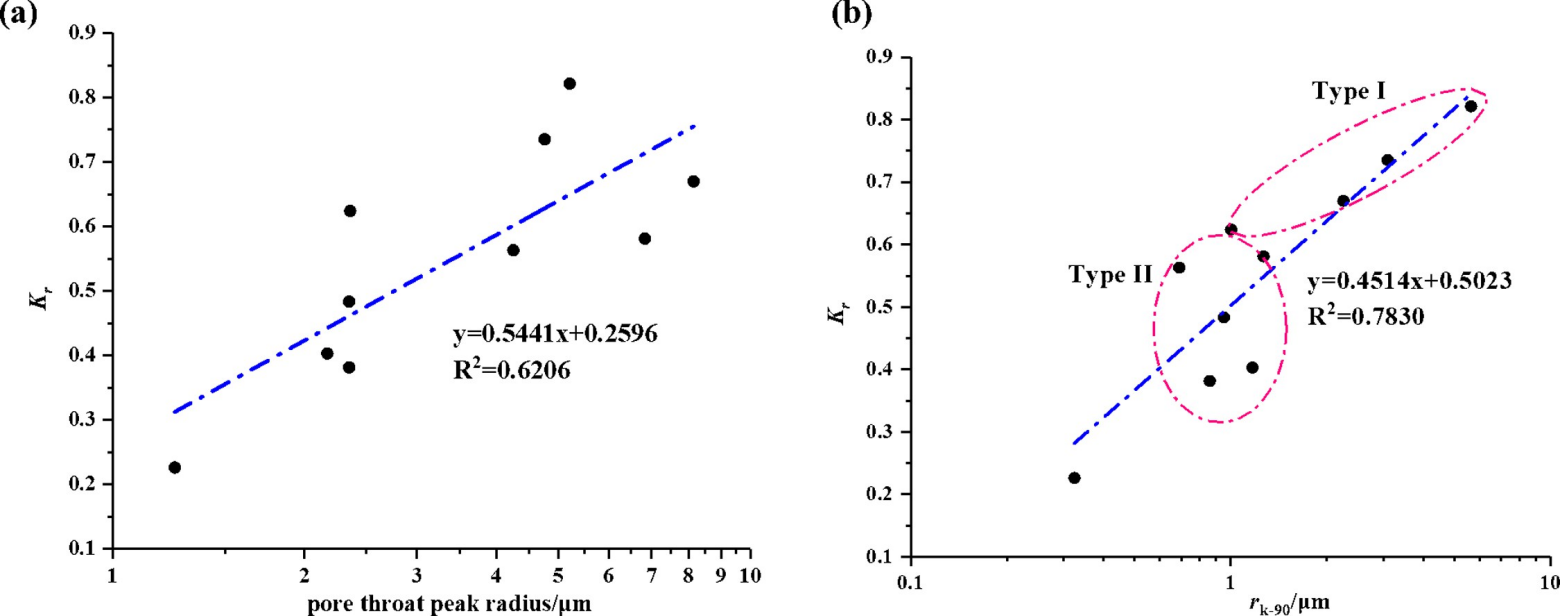
The correlation between the permeability recovery ratios *K*_r_ and the values of *r*_peak_ (a) and *r*_***k-*90**_ (b).

According to section 4.1, we can know that the larger pores mainly contribute to the percolation capacity of core plugs. Herein, the pore throat peak radius (*r*_peak_) and the pore radius corresponding to the 90% of cumulative permeability contribution (*r*_*k*-90_) were used to assess the influence of PSD on water blocking damage. Fig 11(A) shows a positive correlation between the values of *r*_peak_ and the permeability recovery ratios *K*_r_ with a correlation coefficient of 0.6206. It means that the permeability of core sample with a larger *r*_peak_ can more easily increase after the gas flooding. Note that there are some outlier spots around the fitted line. Therefore, the *r*_peak_ is inadequate to evaluate the influence of pore structure on water trapping damage.

The *K*_r_ and *r*_*k*-90_ for all samples were plotted in Fig 11(B). It is evident that a positive correlation was observed between the permeability recovery ratios *K*_r_ and the *r*_*k*-90_ by linear fitting. However, it is worth noted that this correlation displays strong for the core samples of type I, whereas the relation exhibits a weak correlation for the samples of type II. This may be explained by the fact this relation can be found obviously only in a wide range. For the core samples of type II, the values of *r*_*k*-90_ fall in a narrow range, so it is difficult to distinguish the difference between different cores by the values of *r*_*k*-90_.

**Fig 12:**
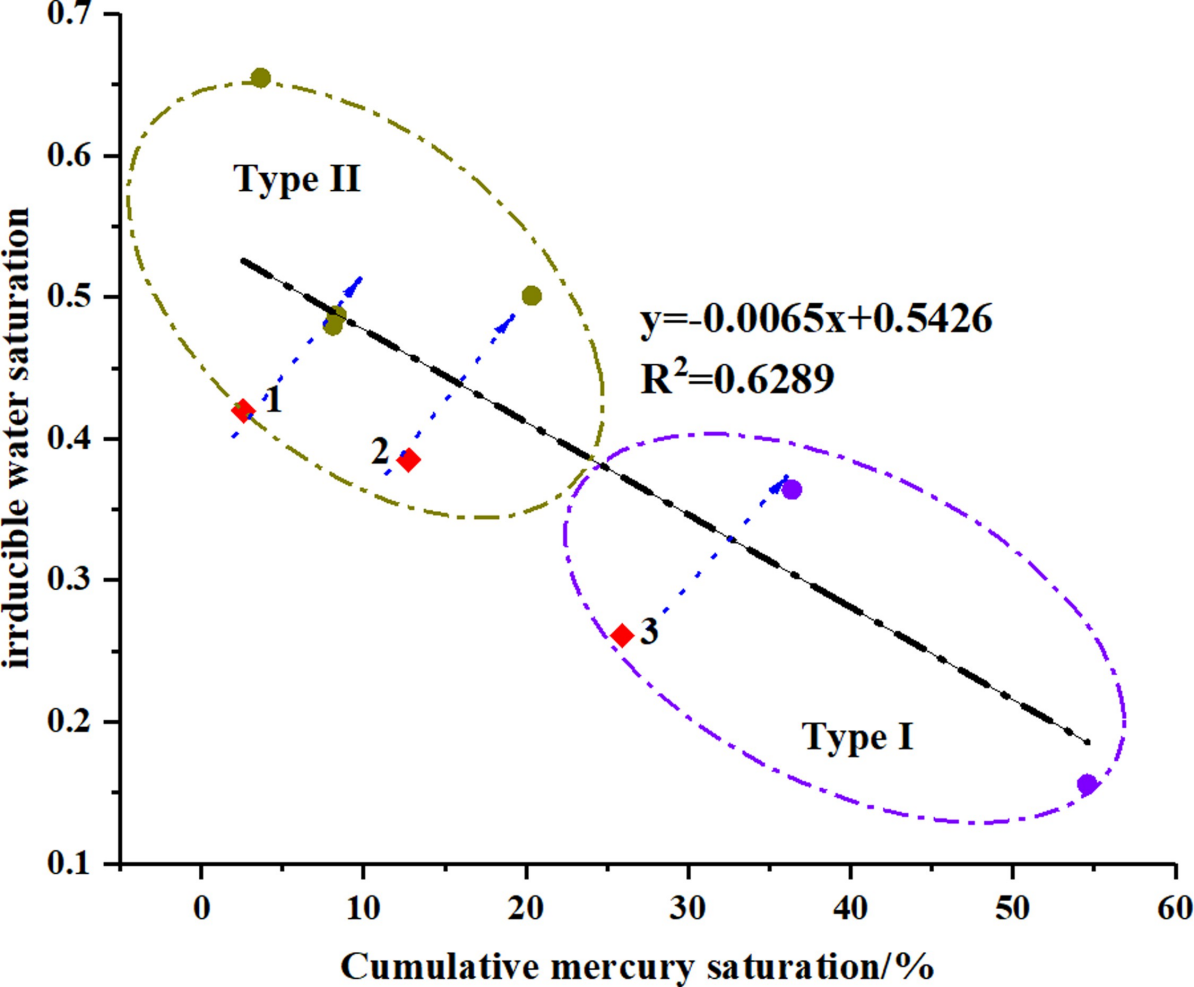
The correlation between *S*_k-90_ and irreducible water saturation.

Moreover, the correlation between *S*_k-90_ and irreducible water saturation shown in Fig 7. An implication of the *S*_k-90_ is the possibility that the related pore spaces over *S*_k-90_ dominated the percolation capacity, while the rest controlled the storage capacity. For instance, for the sample QL-21#, 25.92% of pore volume dominated the 90% of cumulative permeability, and thus 74.08% of pore space nearly makes no contribution to seepage capacity. In other words, once fluids intrude into this pore space, fluids would be trapped and become irreducible liquids. Fig 12 compares the relationship between the values of *S*_k-90_ and irreducible water saturations, a negative correlation between them. It means that a core sample with larger *S*_k-90_ exists less pores to confine fluids, implying the lower irreducible water saturation after the drainage process. Note here that three outlier spots exhibit opposite correlations, which lead to the relatively low correlation coefficients of the linear fitting. The outliers would be discussed in the following section. Peng et al. [51] roughly divided the dataset into three clusters according to the *S*_*wir*_ values, and they supported that for samples with the same porosity, the *S*_*wir*_ values can reflect the pore sizes and structures, which is, the *S*_*wir*_ values become lower with the higher permeability. This offers hints about the influence of similar parameters like dominant pore sizes, pore connectivity and pore structures on *S*_*wir*_ and permeability.

Overall, it is appropriate to say that the parameters obtained from pore size distributions can partly reflect the influence of pore structure on the water blocking damage, whereas there could be other factors which are related to this formation damage.

### 4.3 Relation between the fractal dimensions and phase trapping damage

**Fig 13:**
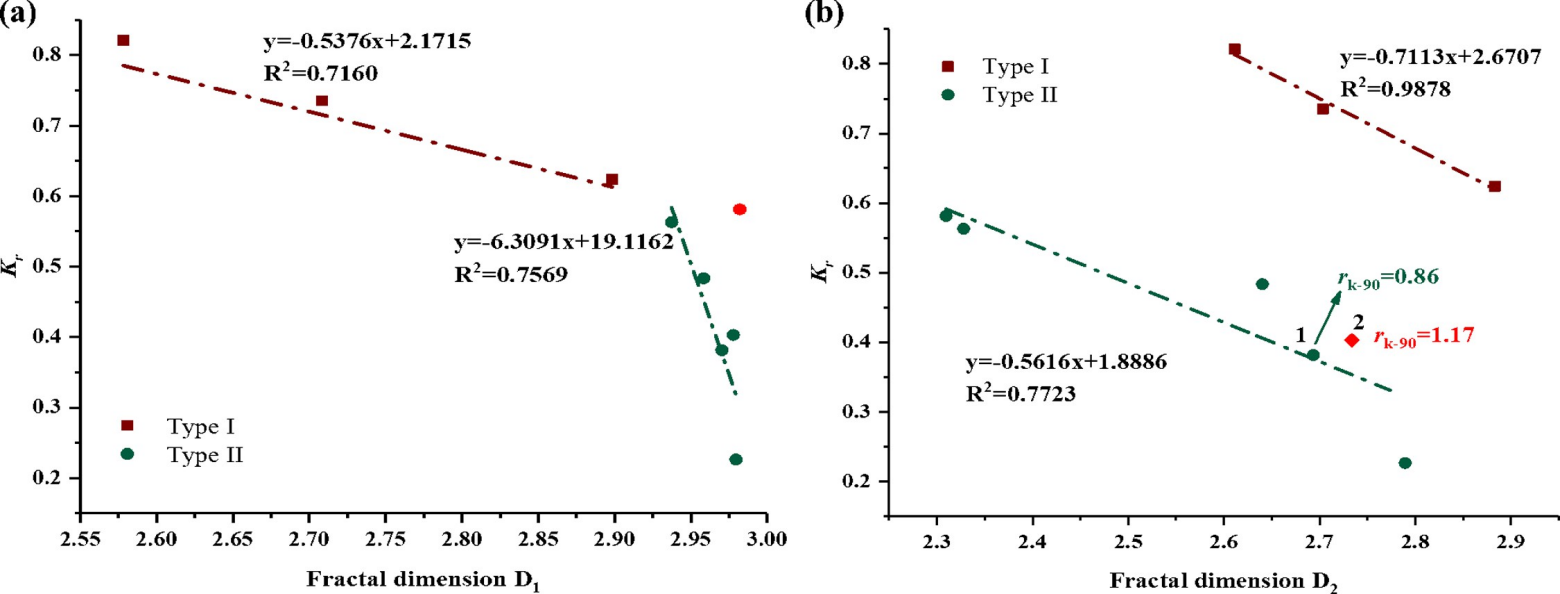
The relationship between the permeability recovery ratios *K*_r_ and fractal dimensions (a): D_1_; (b) D_2_.

Now we have known that the PSD exerted an essential influence on water blocking damage, whereas some outliers implied that the PSD is inadequate in clarifying this damage. Herein, we further analyzed the relationship between the fractal dimensions of core samples and the phase trapping damage. Fig 13(A) and 13(B) plot the *K*_r_ versus D_1_ and D_2_, respectively. The results revealed that the *K*_r_ correlated with D_1_ and D_2_ for the 9 samples. However, unlike the relationship between PSD and *K*_r_, the relationships between fractal dimensions and *K*_r_ are clearly divided into two regions based on the core types, showing a distinct discrepancy between the core samples of the two types. Fig 13A shows that a good negative correlation can be found between the fractal dimensions D_1_ and *K*_r_ for the samples of type I, meaning that a higher heterogeneity leads to a decrease in percolation capacity, while for the samples of type II, D_1_ exhibited a relatively weaker correlation with *K*_r_. If the red spot, an outlier, had been excluded in the linear fitting, the correlation coefficient would reach to around 0.9. Note that the D_1_ of samples of type II were in a narrow range of 2.9 to 3, and thus we may say that, for the samples of type II, the large-pores section exhibited a poor fractal characteristic. Here, it should be emphasized that fractals characterize a self-similar geometry at a certain scale of magnification, which means that a fractal object exhibits similar patterns at increasingly small scales [44, 45, 52]. However, for the samples of type II, the majority of pore characterized in relatively smaller sizes, and the fraction of macropores are limited and asymmetric. Therefore, the D_1_ values of core samples of type II should be used with caution to predict pore structure and APT damage in tight gas sandstone due to the narrow range of D_1_ values. As for D_2_ for all samples, the values fall in a scope of 2.3096 to 2.8833 without an abnormally high data, meaning that the smaller pores segments were in accordance with the fractal distributions. As we can see, the D_2_ for samples of type I and type II exhibited a good negative relation with *K*_r_ with a correlation coefficient of 0.9878 and 0.7723, respectively. The results indicates that for those core samples with very close permeability, the complexity and heterogeneity of flow channels in pores becomes the determining factor for the flow capacity. The significance of this finding is that for tight gas sandstone, fractal dimensions, especially the small pore fractal dimension (D_2_), can be used to predict the possible APT damage very well.

Nevertheless, we noticed that the spot 2, marked in red, deviated from the positive correlation. Spot 2 has a higher value of fractal dimension D_2_ than spot 1, thus the *K*_r_ of spot 2 should be less than that of spot 1 based on the above discussion. However, as we can see, it turned out to be just the opposite. This inconsistency may be due to the fact that the *r*_k-90_ of spot 2 shows higher than that of spot 1. Although the *K*_r_ for samples of type II exhibited a weak correlation with *r*_k-90_ according to the section 4.3, the significantly larger parameters *r*_k-90_ describing pore size can still reasonably explain the reason of this outlier. Therefore, it is appropriate to say that the pore size and fractal characteristic jointly dominate the percolation capacity of tight sandstone.

**Fig 14:**
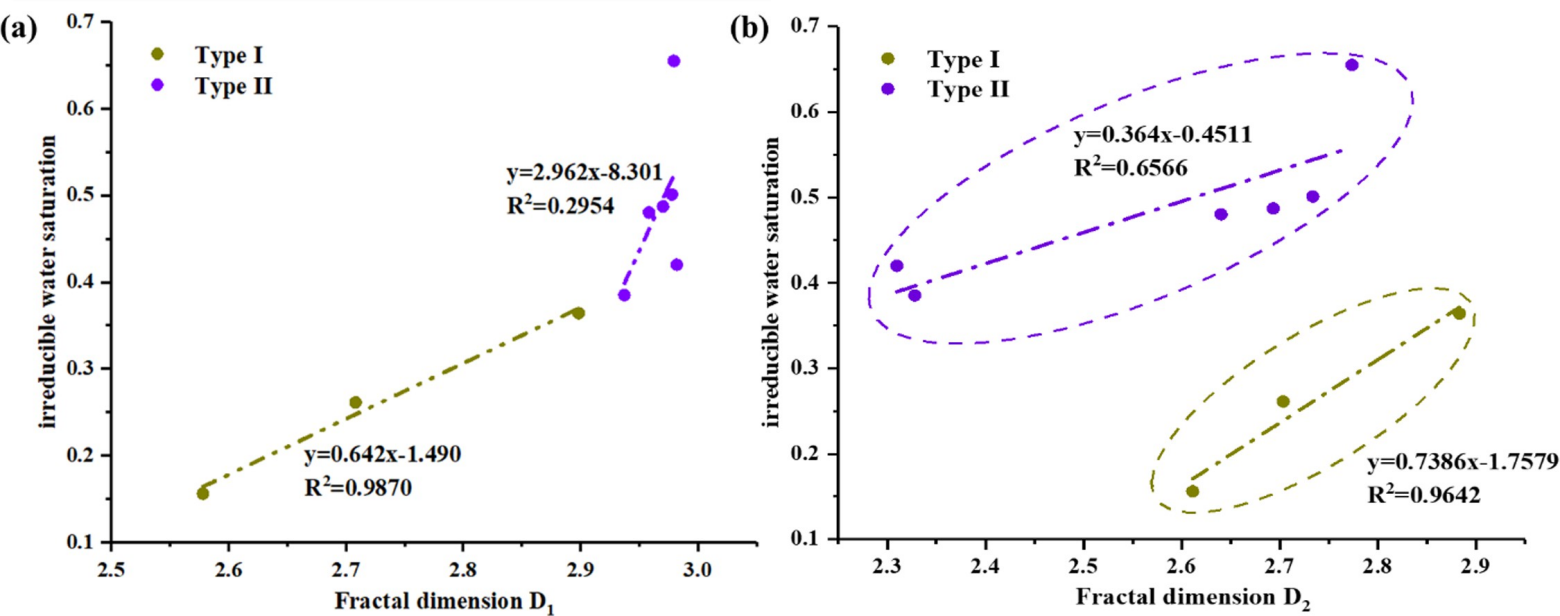
The relationship between fractal dimensions and irreducible water saturations.

The fractal dimensions and irreducible water saturations were plotted in Fig 14. It is evident that the spots were scattered in two different subsets which correspond to the PSD types of samples. For all core samples, the D_2_ correlated well with irreducible water saturations, which was agreement in the previous studies that the flow capacity of tight sandstone was mainly controlled by D_2_.

According to the section 4.3, the irreducible water saturations were also affected by the *S*_k-90_ excepted three abnormal points. In fact, when we look at the D_2_ of the three abnormal points, we can know that they have relatively low fractal dimensions of D_2_, which can reduce the irreducible water saturations. It supports the aforementioned conclusion that the small pore fractal dimension (D_2_) can be employed in conjunction with the aperture parameter for more accurate APT prediction.

## 5. Conclusion

1. The porosity of core samples in the Ordos Basin ranges from 5.68% to 13.7%, with an average of 9.52%, and the permeability ranges from 0.00456 to 7.86 mD (with an average of 1.272 mD), which is a typical tight reservoir with strong heterogeneity. Quartz mineral is the main component of sandstone in Ordos Basin. Four types of pores are identified in the core samples in tight sandstone: primary intergranular pores, dissolution intergranular pores, dissolution intragranular pores and intercrystalline pores.
2. According to the HPMI capillary curve, the cores can be divided into two types: Type I and Type II. The *r*_max_ of type 1 ranges from 2.356 μm to 8.212 μm, and the average pore size ranges from 0.577 μm to 1.7696 μm. The *r*_max_ of type II ranges from 0.0383 to 2.6430 μm, and the average pore ranges from 0.1029 to 0.3564 μm. The pore throats of type I are larger than that of type II. The pore structures of tight sandstones display the multifractal fractal feature: D_1_ corresponding to macro-pores, and D_2_ corresponding to fractal dimension of micro-pores.
3. Pore size and fractal characteristic jointly dominate the percolation capacity of tight sandstone. Positive correlations between pore size parameters (*r*_*k*-90_ and *r*_peak_) and the permeability recovery ratios *K*_r_ were observed with relatively weak correlations, indicating that PSD is inadequate in clarifying this damage. The *K*_r_ also well correlated with D_1_ and D_2_ for the 9 samples with a distinct discrepancy between the samples of the two types. For those core samples with very close permeability, fractal dimensions, especially the small pore fractal dimension (D_2_), can be used to predict the possible APT damage very well.

In this paper, HPMI data is used to investigate the fractal characteristics of the core samples, but there is a limitation that it is difficult to measure the pores with extremely small pore sizes at the nanoscale due to the limited injection pressure. NMR technology is a gradually widely used technology to characterize the full range of pore size distribution. In future studies, HPMI and NMR technology can be combined to further accurately describe the pore size distribution and fractal dimension of tight gas sandstone, so as to provide a more comprehensive and accurate prediction method for APT damage of tight gas sandstone.

## Supporting information

S1 Dataset (RAR)
